# Circadian sleep/wake-associated cells show dipeptide repeat protein aggregates in C9orf72-related ALS and FTLD cases

**DOI:** 10.1186/s40478-019-0845-9

**Published:** 2019-12-02

**Authors:** Lieselot Dedeene, Evelien Van Schoor, Rik Vandenberghe, Philip Van Damme, Koen Poesen, Dietmar Rudolf Thal

**Affiliations:** 1 Department of Neurosciences, Laboratory for Molecular Neurobiomarker Research, KU Leuven (University of Leuven), Leuven Brain Institute (LBI), Leuven, Belgium; 2 Department of Imaging and Pathology, Laboratory for Neuropathology, KU Leuven (University of Leuven), Leuven Brain Institute (LBI), Leuven, Belgium; 3 Laboratory Medicine, University Hospitals Leuven, Leuven, Belgium; 4 Department of Neurosciences, Laboratory for Neurobiology, KU Leuven (University of Leuven) and Center for Brain & Disease Research, VIB, Leuven Brain Institute (LBI), Leuven, Belgium; 5 Department of Neurology, University Hospitals Leuven, Leuven, Belgium; 6 Department of Neurosciences, Laboratory for Cognitive Neurology, KU Leuven, Leuven Brain Institute (LBI), Leuven, Belgium; 7 Department of Pathology, University Hospitals Leuven, Leuven, Belgium

**Keywords:** Amyotrophic lateral sclerosis, Frontotemporal dementia, C9orf72, Dipeptide repeat proteins, TDP-43, Pineal gland, Circadian rhythm, Sleep/wake cycle

## Abstract

Motor-, behavior- and/or cognition-related symptoms are key hallmarks in patients with amyotrophic lateral sclerosis (ALS) and frontotemporal lobar degeneration (FTLD) with TDP-43 pathology (FTLD-TDP), respectively. It has been reported that these patients also experience sleep disturbances, which might implicate a disturbed circadian rhythm of the sleep/wake cycle. It remains unknown, however, whether cells involved in the circadian sleep/wake cycle are affected by ALS- and FTLD-related neuropathological changes including phosphorylated TDP-43 (pTDP-43) aggregates and dipeptide repeat protein (DPR) inclusions resulting from the *C9orf72* hexanucleotide repeat expansion. Immunohistochemistry for DPR and pTDP-43 pathology was performed in post-mortem hypothalamus and pineal gland tissue of patients with ALS and/or FTLD-TDP with and without the *C9orf72* repeat expansion and healthy controls. Circadian sleep/wake-associated cells, including pinealocytes and hypothalamic neurons related to the suprachiasmatic nucleus (SCN), were microscopically assessed. We observed numerous DPR inclusions (poly(GA), poly(GP), poly(GR) and poly(PR)) in the pinealocytes and few poly(GA) inclusions in the SCN-related neurons in *C9orf72-*related ALS and/or FTLD-TDP cases. These circadian sleep/wake-associated cells, however, were devoid of pTDP-43 pathology both in *C9orf72-* and non*C9orf72-*related ALS and/or FTLD-TDP cases. Our neuropathological findings show that pinealocytes and, to a lesser extent, SCN-related neurons are affected by DPR pathology. This may reflect an involvement of these cells in sleep/wake disturbances observed in ALS and/or FTLD-TDP patients.

## Introduction

The hexanucleotide (GGGGCC) repeat expansion in the chromosome 9 open reading frame 72 (*C9orf72*) gene is the underlying genetic cause in approximately half of the familial amyotrophic lateral sclerosis (ALS) cases and in about 10% of the sporadic ALS cases [18]. Moreover, this *C9orf72* repeat expansion connects ALS to frontotemporal lobar degeneration with transactive response DNA-binding protein 43 kDa (TDP-43) pathology (FTLD-TDP) by representing a quarter of the familial FTLD cases [34]. Patients carrying this *C9orf72* repeat expansion show aberrant protein aggregates in neurons. These protein aggregates represent, on the one hand, dipeptide repeat proteins (DPRs) arising from unconventional repeat-associated non-ATG translation of the *C9orf72* repeat expansion and, on the other hand, TDP-43, a nuclear protein, which is mislocalized to the cytoplasm [30, 31]. Apart from symptoms related to the loss of both upper and lower motor neurons, it has been reported that patients with ALS also experience a disturbed sleep pattern, daytime sleepiness and fatigue [1, 9, 20, 24, 25]. These sleep-related symptoms are still underdiagnosed and are mainly considered as a consequence of muscle weakness and respiratory issues [39]. Patients with FTLD also show sleep/wake disturbances similar to sleep problems in patients with Alzheimer’s disease (AD), although starting earlier in the disease course [2, 28]. One study showed a potential involvement of the *C9orf72* repeat expansion in rapid eye movement sleep behavior disorder (RBD) by identifying two *C9orf72* repeat expansion carriers in a cohort of 344 RBD patients [13]. Moreover, these two RBD patients were carriers of a risk haplotype associated with *C9orf72*-related ALS and FTLD [29]. This suggests that patients with ALS and/or FTLD-TDP carrying the *C9orf72* repeat expansion could be more vulnerable to sleep abnormalities. Nevertheless, studies providing an in-depth characterization of the previously mentioned sleep problems in ALS and/or FTLD-TDP patients with and without the *C9orf72* repeat expansion are yet to be performed.

In other neurodegenerative disorders, including AD and Parkinson’s disease (PD), the sleep/wake cycle is disturbed along with changes in circadian melatonin levels [6, 38, 40, 42]. Whether similar circadian rhythm disturbances are at the root of sleep problems in ALS and/or FTLD-TDP patients remains elusive [1, 24]. In an *SOD1*^G93A^ mouse model of ALS, artificially induced circadian rhythm dysfunction accelerated the disease onset as measured by motor function tests and disease progression in terms of body weight loss [22]. Moreover, this circadian rhythm dysfunction aggravated degeneration of motor neurons in the spinal ventral horn and increased astrocytic and microglial activation [22]. Furthermore, in an ALS/frontotemporal dementia (FTD) rat model bearing a *FUS* point mutation (R521C), the onset of cognitive deficits was preceded by circadian rhythm abnormalities and disturbances in the sleep/wake cycle [41]. Therefore, these findings point to the direction of circadian rhythm disturbances in ALS and FTD.

The two major brain structures regulating the circadian sleep/wake cycle are, on the one hand, the suprachiasmatic nucleus (SCN) (“the central biological clock”) located in the hypothalamus and, on the other hand, the melatonin-producing pineal gland acting as the main executor of the SCN. The SCN suppresses or stimulates the pineal synthesis of melatonin according to the light/dark cycle, leading to a decreased or increased tendency to sleep. In AD cases, neurofibrillary tangle pathology and plaques were observed in the SCN, but not in the pineal gland [32, 36]. In PD cases, Lewy body pathology was observed in the SCN, and rarely in the pineal gland [17]. In ALS and/or FTLD-TDP patients, it remains unknown whether and, if so, which cells involved in the circadian sleep/wake cycle are affected by pathological changes. A better understanding of the underlying pathological mechanism of circadian sleep/wake disturbances may provide new insights in the involvement of this type of disturbances in the disease course of ALS and FTLD. To this end, we immunohistochemically investigated circadian sleep/wake-associated cells (i.e. the pineal gland and SCN-related neurons in the hypothalamus) for the presence of ALS- and FTLD-TDP-related pathological protein inclusions (DPRs and phosphorylated TDP-43 (pTDP-43)) in patients with ALS and/or FTLD-TDP with and without the *C9orf72* repeat expansion.

## Materials and methods

### Human cases

Post-mortem human brain tissue, including the pineal gland and hypothalamus, was provided by the UZ Leuven brain biobank (Belgium) and the municipal hospital Offenbach (Germany) in accordance with the Belgian and German law. This study was approved by the UZ Leuven ethical committee and the UZ Leuven biobank board. Table 1 shows the demographics and tissue availability of the human cases by study groups. A list of the individual human autopsy cases included in this study is provided in Additional file 1. In total, seven ALS and/or FTLD-TDP cases carrying the *C9orf72* hexanucleotide repeat expansion were included (4 ALS, 2 FTLD-TDP and 1 ALS-FTLD). They were further referred to as *C9orf72* cases. The *C9orf72* hexanucleotide repeat expansion was identified by triplet repeat primed PCR on DNA extracted from peripheral blood and/or cerebellum. As comparison for pTDP-43 pathology and as negative controls for DPR pathology, 21 ALS and/or FTLD-TDP cases without the *C9orf72* hexanucleotide repeat expansion were included (11 ALS, 9 FTLD-TDP and 1 ALS-FTLD), further referred to as non*C9orf72* cases. Three healthy controls without neurodegenerative disease were used as negative controls for pTDP-43 pathology. Clinical assessment was performed by an expert neurologist. The diagnosis of ALS was based on the revised El Escorial criteria and the Awaji algorithm [8, 15, 16]. FTLD patients were diagnosed according to published criteria [21, 33]. An experienced pathologist carried out the autopsy. Microscopically, the diagnosis of ALS was assessed by TDP-43 pathology [7, 31]. FTLD-TDP was neuropathologically diagnosed using the Mackenzie criteria [27]. AD and PD pathologies were assessed according to the National Institute on Aging and Alzheimer’s Association (NIA-AA) criteria [23] and the Braak-PD stages [5], respectively. Concomitant AD or PD pathology was absent or mild in all cases (NIA-AA degree of AD pathology 0–1 [23]; Braak-PD stage 0–1 [5]) (Additional file 1).

**Table 1:** Demographic data and tissue availability by study groups

	Male: female^a^	Mean age in years (SD)^b^	Neuro-pathological diagnosis	Number of cases available					Mutations
				Pineal gland	Hypo-thalamus^c^	SCN-related neurons	SON	PVN	
*C9orf72* cases	6:1	56.7 (4.8)	ALS	4	4	3	3	3	*C9orf72*
			FTLD-TDP	2	2	2	2	2	*C9orf72*
			ALS-FTLD	0	1	1	1	1	*C9orf72*
			Total	6	7	6	6	6	/
non*C9orf72* cases	13:8	62.1 (11.2)	ALS	9	11	2	6	7	10 no mutation, 1 *TARDBP*
			FTLD-TDP	7	9	2	6	7	5 no mutation, 1 *TUBA4A,* 1 *GRN,* 1 *VCP,* 1 *TBK1*
			ALS-FTLD	1	1	1	1	1	No mutation
			Total	17	21	5	13	15	/
Healthy control cases	2:1	63.0 (2.7)	Healthy control	3	3	0	0	0	No mutation
			Total	3	3	0	0	0	/

^a^ The sex did not significantly differ between the groups as analyzed by Fisher’s exact test (*C9orf72* vs. non*C9orf72* cases, *p* = 0.37; *C9orf72* vs. healthy control cases, *p* > 0.99; non*C9orf72* vs. healthy control cases, p > 0.99)

^b^ The age did not significantly differ between the three groups as tested by one-way ANOVA (*p* = 0.43)

^c^ This column represents the number of cases with hypothalamus sections available that were screened for the presence of SCN-related neurons, SON and PVN

VIP-ir neurons indicates vasoactive intestinal peptide-immunoreactive neurons; SON, supraoptic nucleus; PVN, paraventricular nucleus; SD, standard deviation

### Immunohistochemistry

Histological examination of the pineal gland and the hypothalamus was performed on 5-μm-thick sections cut from formalin-fixed, paraffin embedded tissue. Primary antibodies used in this study were mouse monoclonal anti-poly(GA) clone 5E9 (MABN889, Merck Millipore, Billerica, USA) at a dilution of 1/1000 for 30 min, rat monoclonal anti-poly(GR) clone 5A2 (MABN778, Merck Millipore) at a dilution of 1/400 overnight, custom-made rabbit poly(GP) (Thermo Scientific, Waltham, USA) [19, 37] at a dilution of 1/1000 for 30 min, custom-made rabbit poly(PR) (Thermo Scientific) [19, 37] at a dilution of 1/50 overnight, mouse monoclonal anti-pTDP-43 (pS409/410) (TIP-PTD-M01, Cosmo Bio, Tokyo, Japan) or rabbit polyclonal anti-pTDP-43 (pS409/410–2) (TIP-PTD-P02, Cosmo Bio) at a dilution of 1/2500 (double immunostainings) or 1/5000 (single immunostainings) for 30 min, mouse monoclonal anti-synaptophysin ready-to-use 1/1 (IR660, Agilent) for 30 min and polyclonal rabbit anti-vasoactive intestinal peptide (VIP) (HPA017324, Sigma-Aldrich, Saint Louis, MO, USA) at a dilution of 1/300 for 30 min. Stainings for poly(GA), poly(GP), poly(GR), poly(PR) and pTDP-43 were performed as described before [19]. In brief, poly(GP) and pTDP-43 immunostainings were automatically performed by means of the BOND-MAX automated staining system (Leica Biosystems, Wetzlar, Germany) using the Bond Polymer Refine Detection kit (DS9800, Leica Biosystems). Immunohistochemistry for poly(GA) was partially performed in the BOND-MAX automated staining system. Poly(GR) and poly(PR) were performed fully manually. Low pH heat pretreatment was used for all antibodies except for anti-synaptophysin. For the latter, high pH heat pretreatment was used. For poly(GA) and poly(GR) immunostaining, an additional pretreatment with formic acid was performed to enhance the signal. Since endogenous brown colored material of the pineal gland tissue interfered with the analysis of small poly(GP), poly(GR), poly(PR) and pTDP-43 inclusions visualized by 3,3′-diaminobenzidine (DAB), these inclusions were also visualized by a Fast Red-type chromogen using the Dako REAL Detection System (K5005, Agilent, Santa Clara, CA, USA) for poly(GR) and poly(PR) or the Bond Polymer Refine Red Detection kit (DS9800, Leica Biosystems) for poly(GP) and pTDP-43. Double immunostainings were performed using the BOND-MAX automated staining system. For the double staining of synaptophysin and DPRs, the pineal gland of three *C9orf72* cases and two non*C9orf72* cases was first stained with poly(GA) or poly(GP) visualized by DAB (high pH pretreatment). Afterwards, synaptophysin immunostaining was visualized by Fast Red. For the double immunostainings of VIP and poly(GA) or pTDP-43, VIP immunostaining was performed first and visualized by DAB (low pH pretreatment). Afterwards, a second low pH heat pretreatment and poly(GA) (with additional formic acid pretreatment) or pTDP-43 immunostaining was performed and visualized by Fast Red.

### Microscopic assessment

The VIP-immunoreactive (ir) neurons evaluated for DPR and pTDP-43 pathology were located in the hypothalamus in between the supraoptic nucleus (SON) and paraventricular nucleus (PVN). This VIP-ir area covers (relay) neurons and efferent projections related to the suprachiasmatic nucleus (SCN) [12]. Consequently, these VIP-ir neurons are presumably involved in sleep/wake circadian rhythm regulation and were further referred to as SCN-related neurons. The pinealocytes and the magnocellular cells of the SON and PVN were neuroanatomically identified by their morphological pattern. The aforementioned brain regions were not available in all cases due to limited sample availability (Table 1, Additional file 1). DPR and pTDP-43 pathologies were assessed by two separate investigators. The assessment of pTDP-43 pathology was performed blinded to the diagnosis and genetics of the patients. DPR pathology was assessed unblinded, since *C9orf72* cases show abundant DPR pathology and non*C9orf72* cases do not show DPRs at all. This characteristic staining pattern precludes a blinded evaluation. Evaluation of DPR and pTDP-43 pathology in the pineal gland (*C9orf72 n* = 6, non*C9orf72 n* = 17, healthy control *n* = 3), SON (*C9orf72* n = 6, non*C9orf72 n* = 13) and PVN (*C9orf72 n* = 6, non*C9orf72 n* = 15) was performed using a semiquantitative grading system, adapted from a previously published grading system [19]. The total amount of pathology was counted in a 40x visual microscopic field with most abundant pathology, considered as the “hotspot area”. DPR and pTDP-43 pathology was rated as ‘0’ if no pathology was present, as ‘1’ if 1 to 5 pathological lesions were present, as ‘2’ if 6 to 20 pathological lesions were present, as ‘3’ if 21 to 50 pathological lesions were present and as ‘4’ if more than 50 pathological lesions were present in the hotspot area. To evaluate pathology in the SCN-related neurons (*C9orf72 n* = 6, non*C9orf72 n* = 5), the number of VIP-ir neurons containing poly(GA) or pTDP-43 pathology was divided by the total number of VIP-ir neurons observed in the aforementioned area of the hypothalamus section. Per case, 1 to 13 VIP-ir neurons were observed. The available hypothalamus sections of the healthy control cases did not contain VIP-ir neurons (Table 1). The Leica DM2000 LED microscope (Leica Biosystems) coupled to a Leica DFC 7000 T camera was used. Images were processed in ImageJ software and combined into figures using CorelDRAW.

### Statistical analysis

Statistical analysis was performed with GraphPad Prism 8.0.1. To compare the age and sex between the groups, a One-way ANOVA test and a Fisher’s exact test were used, respectively. Pathological assessments in the *C9orf72* and the non*C9orf72* ALS and/or FTLD-TDP cases were compared by a Mann-Whitney test. The significance level was set at 5%.

## Results

### Abundant DPR pathology in the pineal gland of *C9orf72* cases

**Fig. 1:**
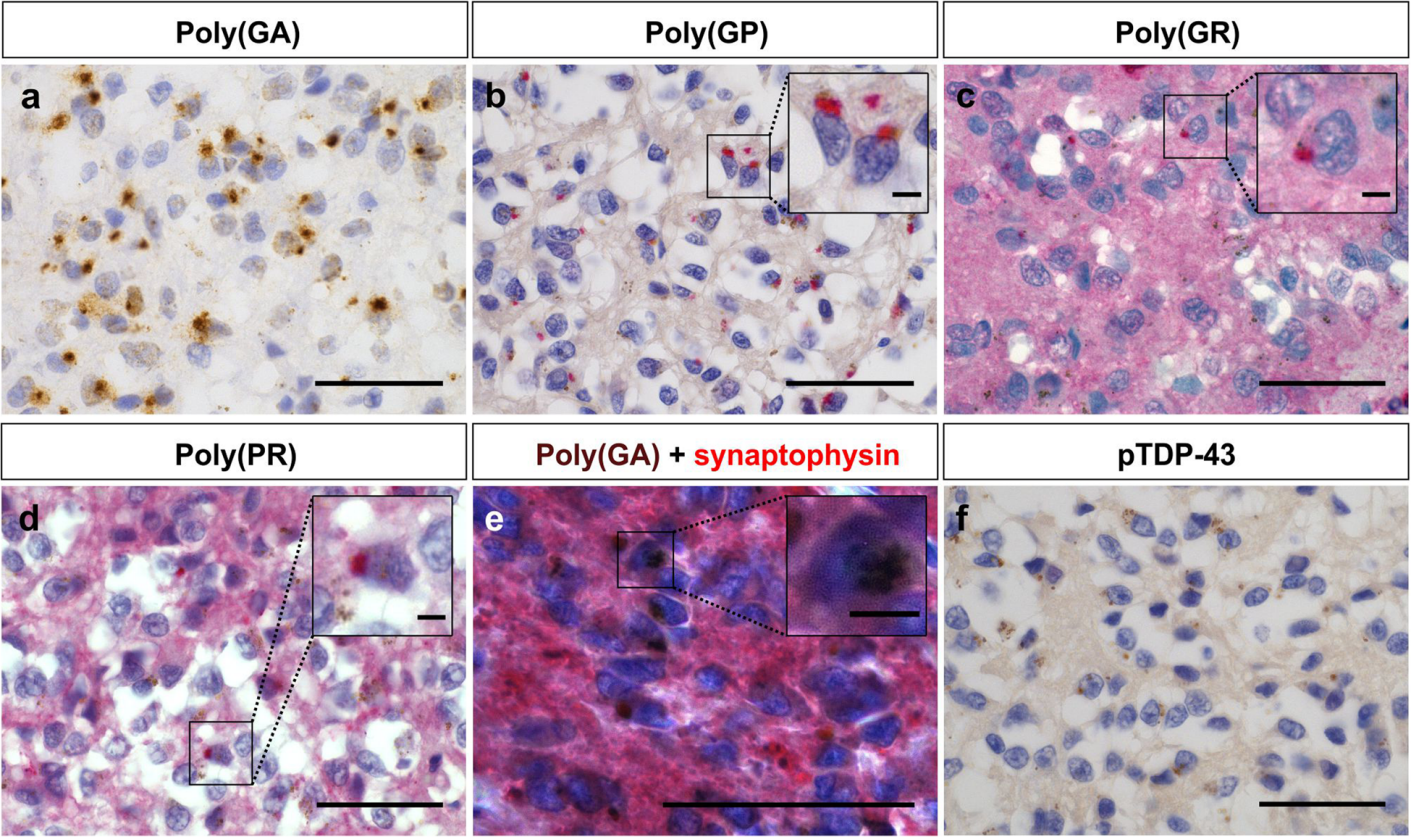
DPR and pTDP-43 pathology in the pineal gland of *C9orf72* and non*C9orf72* cases. Poly(GA) (**a**) and poly(GP) (**b**) inclusions were observed in the pineal gland of all *C9orf72* cases. To a lesser extent, poly(GR) (**c**) and poly(PR) (**d**) inclusions were present in the pineal gland of *C9orf72* cases. Panels **a-d** show immunohistochemical stainings of case C9–7. The insets show a magnification of the respective DPR inclusions. The chromogen used for visualization of poly(GP), poly(GR) and poly(PR) inclusions is a Fast Red-type chromogen, therefore, a red background color was obtained. Poly(GA) inclusions (visualized by DAB) are present in the melatonin-producing pinealocytes expressing synaptophysin (visualized by Fast red) shown here in case C9–1 (**e**). The **inset of e** shows a magnification of a synaptophysin-positive neuron with a poly(GA) inclusion. The pineal gland is devoid of pTDP-43 pathology (visualized by Fast red) (**f**). Endogenous brown colored material was observed in the tissue (**b-d, f**). Scale bar represents 50 μm, the scale bar of the insets represents 5 μm. DAB stands for 3,3′-diaminobenzidine

The neuropathological diagnosis, sex and age by study groups is shown in Table 1 and a list of the individual cases included in this study is provided in Additional file 1. To investigate the ALS and FTLD-TDP-related pathological changes of the melatonin-producing brain structure, the pineal gland of six *C9orf72* cases and 17 non*C9orf72* cases was analyzed for DPR and pTDP-43 pathology (Table 1-2, Additional file 1). DPR pathology was observed in the pineal gland of all *C9orf72* cases (*p* < 0.0001) (Fig. 1a-d, Table 2, Additional file 1), more specifically in the melatonin-producing pinealocytes as identified by synaptophysin expression (Fig. 1e). The pineal gland of non*C9orf72* and healthy control cases was negative for DPR pathology (Table 2, Additional file 1, Additional file 2: Figure S1). The relative abundance of the distinct DPR species in the pineal gland of *C9orf72* cases was similar as in other brain regions (poly(GA) > poly(GP) > poly(GR) > poly(PR)), as previously quantified [19] (Table 2, Additional file 1). In all *C9orf72*, non*C9orf72* and healthy control cases, the pineal gland was virtually free of pTDP-43 pathology (Fig. 1f, Table 2, Additional file 1). As such, no differences in pTDP-43 pathology were observed in the pineal gland sections of *C9orf72* cases compared to non*C9orf72* cases.

**Table 2:** Neuropathological analysis of the investigated brain regions

	*C9orf72* ALS and/or FTLD-TDP cases		non*C9orf72* ALS and/or FTLD-TDP cases		Healthy control cases	
Pineal gland	Number of positive cases	Median score (IQR, range)	Number of positive cases	Median score (IQR, range)	Number of positive cases	Median score (IQR, range)
Poly(GA)	6/6 (100%) (p < 0.0001)^a^	4 (1; 2–4)	0/17 (0%)	0 (0; 0–0)	0/3 (0%)	0 (0; 0–0)
Poly(GP)	3/3 (100%) 3 n.a.^c^	4 (3; 1–4)	n.a.^d^	n.a.^d^	n.a.^d^	n.a.^d^
Poly(GR)	3/3 (100%) 3 n.a.^c^	1 (1; 1–2)	n.a.^d^	n.a.^d^	n.a.^d^	n.a.^d^
Poly(PR)	2/3 (67%) 3 n.a.^c^	1 (1; 0–1)	n.a.^d^	n.a.^d^	n.a.^d^	n.a.^d^
pTDP-43	0/6 (100%) ^b^	0 (0; 0–0)	0/17 (0%)	0 (0; 0–0)	0/3 (0%)	0 (0; 0–0)
Hypothalamus	Number of positive cases	Median score (IQR, range)	Number of positive cases	Median score (IQR, range)	Number of positive cases	Median score (IQR, range)
Poly(GA)	7/7 (100%)	n.a.^e^	0/21 (0%)	n.a.^e^	0/3 (0%)	n.a.^e^
pTDP-43	4/7 (57%)	n.a.^e^	15/21 (71%)	n.a.^e^	0/3 (0%)	n.a.^e^
SCN-related neurons	Number of positive cases	Mean % (SD) VIP-ir neurons affected	Number of positive cases	Mean % (SD) VIP-ir neurons affected	Number of positive cases	Mean % (SD) VIP-ir neurons affected
Poly(GA)	3/6 (50%) (*p* = 0.1818) ^a^	8.5 (10.6)	0/5 (0%)	0.0 (0.0)	n.a.	n.a.
pTDP-43	0/6 (0%) ^b^	0.0 (0.0)	0/5 (0%)	0.0 (0.0)	n.a.	n.a.
SON magno-cellular cells	Number of positive cases	Median score (IQR, range)	Number of positive cases	Median score (IQR, range)	Number of positive cases	Median score (IQR, range)
Poly(GA)	0/6 (0%) ^b^	0 (0; 0–0)	0/13 (0%)	0 (0; 0–0)	n.a.	n.a.
pTDP-43	0/6 (0%) ^b^	0 (0; 0–0)	0/13 (0%)	0 (0; 0–0)	n.a.	n.a.
PVN magnocellular cells	Number of positive cases	Median score (IQR, range)	Number of positive cases	Median score (IQR, range)	Number of positive cases	Median score (IQR, range)
Poly(GA)	0/6 (0%) ^b^	0 (0; 0–0)	0/15 (0%)	0 (0; 0–0)	n.a.	n.a.
pTDP-43	0/6 (0%) ^b^	0 (0; 0–0)	0/15 (0%)	0 (0; 0–0)	n.a.	n.a.

^a^ *p*-values of Mann-Whitney test are shown for comparison of poly(GA) pathology between the *C9orf72* and the non*C9orf72* ALS and/or FTLD-TDP cases as a reference

^b^ No statistical analysis was performed for pTDP-43 pathology, since the pineal gland and SCN-related neurons of *C9orf72* and non*C9orf72* cases do not show any pTDP-43 pathology. We neither performed statistical analysis for pathological assessments in the SON and PVN, since all cases were negative for poly(GA) and pTDP-43 pathology in the magnocellular cells of these brain nuclei

^c^ Three out of the six *C9orf72* ALS and/or FTLD-TDP cases were not assessed for poly(GP), poly(GR) and poly(PR) pathology since prolonged fixation times of the pineal gland masked the detection of the aforementioned DPR inclusions

^d^ Pineal gland tissue of the non*C9orf72* ALS and/or FTLD-TDP and healthy control cases was only stained for the most abundant DPR (poly(GA)) to confirm the cases did not carry the *C9orf72* repeat expansion

^e^ The general pathology in the hypothalamus was not semiquantitatively assessed

n.a. indicates not available; SCN, suprachiasmatic nucleus; SON, supraoptic nucleus; PVN, paraventricular nucleus; IQR, interquartile range; SD, standard deviation

### Poly(GA) inclusions in SCN-related neurons of *C9orf72* cases

**Fig. 2:**
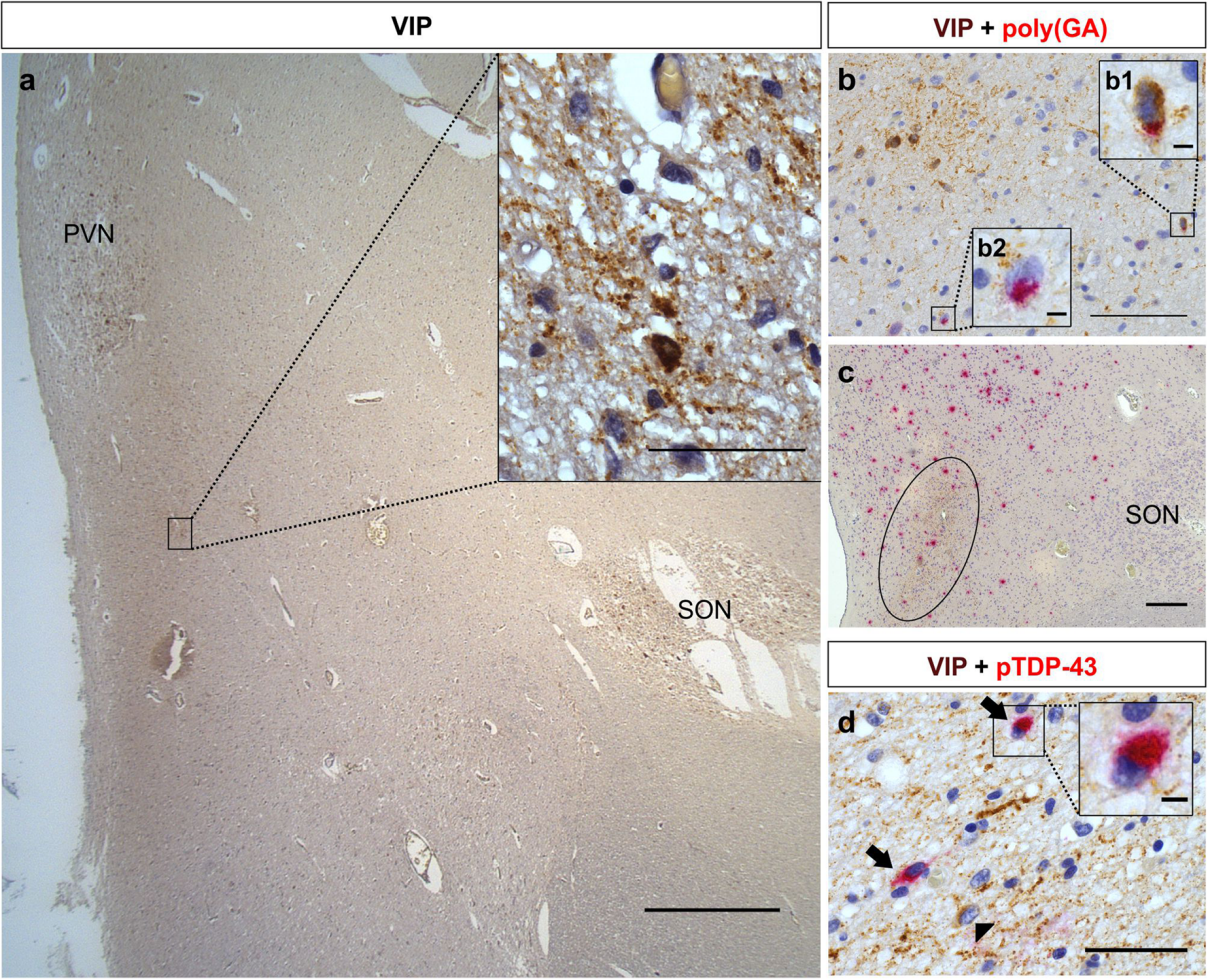
Neuropathological investigation of the SCN-related neurons in the hypothalamus of *C9orf72* and non*C9orf72* cases. VIP-ir neurons in the hypothalamus at the level of the SON and PVN were referred to as SCN-related neurons (case nonC9–16) (**a**). The inset shows VIP-immunostaining in neurons and fibers (**a**). Poly(GA) pathology (visualized by Fast red) was observed in few VIP-ir SCN-related neurons (visualized in brown with DAB) in half of the *C9orf72* cases (case C9–3) (**b**). **Inset b1** shows a magnification of a SCN-related neuron with poly(GA) pathology; **inset b2** shows a magnification of a VIP-negative neuron with a poly(GA) inclusion. Poly(GA) inclusions (visualized by Fast red) were observed in the area of the SCN-related neurons and fibers (encircled) visualized in brown by DAB (**c**), however, more poly(GA) inclusions are observed in the area surrounding the SCN-related neurons and fibers (case C9–1) (**c**). SCN-related neurons were usually spared from pTDP-43 pathology. In some cases, pTDP-43 pathology (black arrows) (visualized by Fast red) was observed in VIP-negative neurons located in between the SCN-related neurons (arrowhead) and fibers (case nonC9–1) (**d**). The **inset in d** shows a magnification of a VIP-negative neuron with pTDP-43 pathology. Scale bars represent 1000 μm in **a**, 100 μm in **b**, 200 μm in **c**, 50 μm in **d** and **inset of a**, and 5 μm in **insets of b and d**. VIP-ir stands for vasoactive intestinal peptide-immunoreactive; SCN, suprachiasmatic nucleus; DAB, 3,3′-diaminobenzidine

In order to evaluate neurons associated with the regulation of the circadian sleep/wake cycle, the SCN-related neurons immunostained for VIP in the hypothalamus sections of six *C9orf72* cases and five non*C9orf72* cases were investigated (Fig. 2a, Table 1, Additional file 2: Figure S2). Poly(GA) - the most abundant DPR – and pTDP-43 pathologies were analyzed in this VIP-ir region by means of a double immunostaining using two distinct chromogens (DAB and Fast Red chromogen to visualize VIP and poly(GA)/pTDP-43, respectively). In 50% of the *C9orf72* cases, 9.1–25.0% of the SCN-related neurons showed poly(GA) pathology (Fig. 2b, Table 2, Additional file 1). In the other half of the *C9orf72* cases and in all non*C9orf72* cases, no poly(GA) pathology in SCN-related neurons was observed (Table 2, Additional file 1). Compared to the absence of poly(GA) pathology in the SCN-related neurons of five non*C9orf72* ALS or FTLD cases, the number of poly(GA)-positive SCN-related neurons in *C9orf72* cases did not reach significance (*p* = 0.1818) (Table 2). Furthermore, in all *C9orf72* cases, VIP-negative neurons in the VIP-ir area showed poly(GA) inclusions (Fig. 2b-c). However, VIP-negative neurons in the VIP-ir area were less affected compared to those in the area surrounding the SCN-related neurons and fibers (Fig. 2c). SCN-related neurons were devoid of pTDP-43 inclusions in all analyzed cases (Fig. 2d, Table 2, Additional file 1), indicating that there is no difference in pTDP-43 pathology in SCN-related neurons between *C9orf72* and non*C9orf72* cases. Due to the absence of pTDP-43 pathology in the VIP-ir neurons, comparison to SCN-related neurons in healthy control cases negative for pTDP-43 pathology was not necessary. In two of the six *C9orf72* cases and two of the five non*C9orf72* cases, VIP-negative neurons in between the SCN-related neurons were affected by pTDP-43 pathological lesions (Fig. 2d).

### No neuropathological changes in the neuroendocrine magnocellular cells of the PVN and SON

**Fig. 3:**
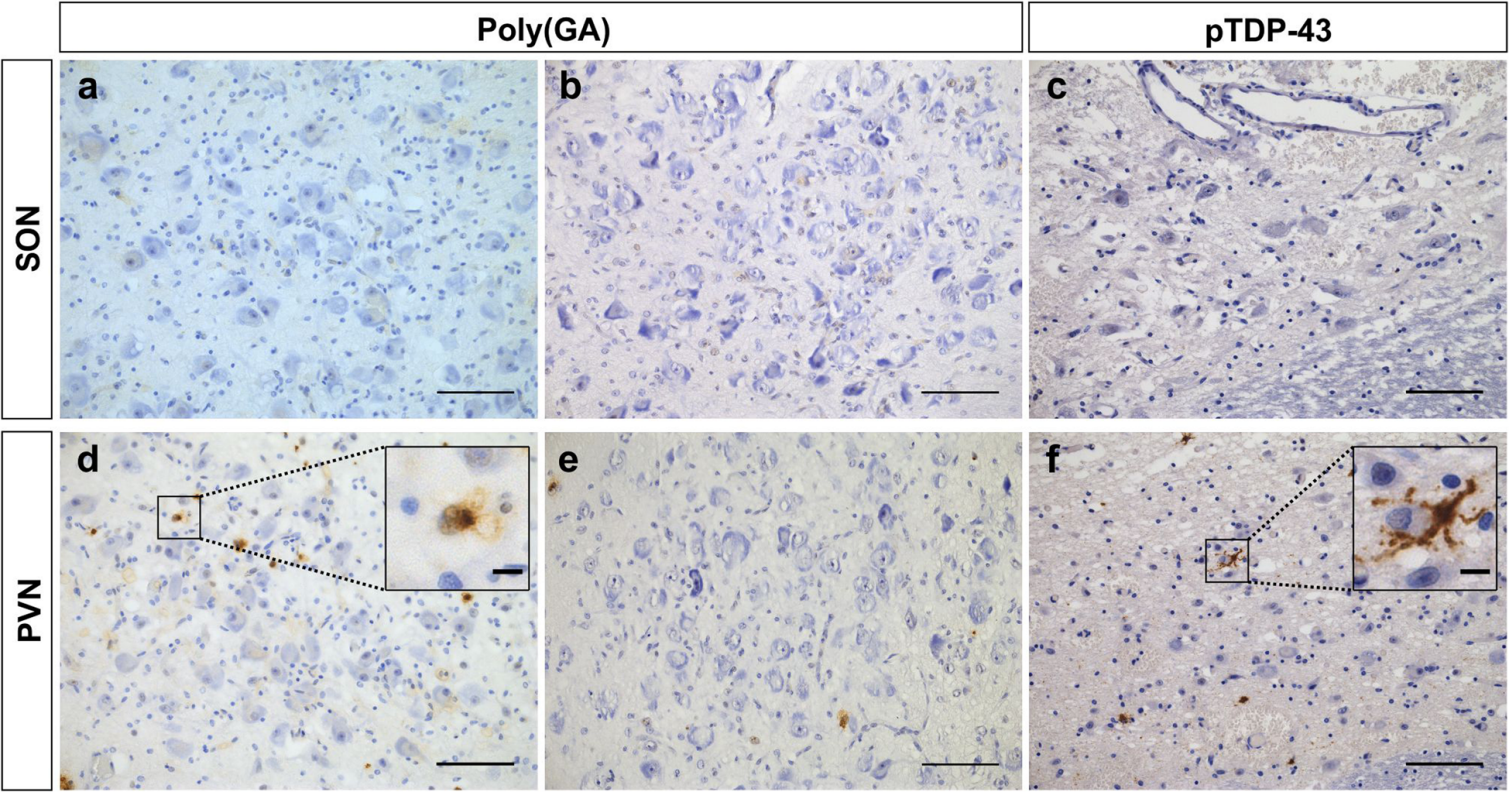
Microscopic assessment of the neuroendocrine magnocellular cells of the SON and PVN in the hypothalamus. The magnocellular cells of the SON (**a-c**) and PVN (**d-f**) were spared from poly(GA) pathology in *C9orf72* cases (case C9–5 and C9–2) (**a-b, d-e**) and devoid of pTDP-43 pathology in *C9orf72* and non*C9orf72* cases (case nonC9–21) (**c,f**). However, pathological lesions appeared in the smaller neurons in between the magnocellular neurons (**d-f**). The **insets in d and f** show a magnification of smaller neurons with poly(GA) and pTDP-43 pathology, respectively. Scale bars represent 100 μm in **a-f** and 10 μm in **the insets of d and f**. SON stands for supraoptic nucleus; PVN, paraventricular nucleus

To compare the vulnerability of the pinealocytes to abnormal protein aggregation with other neuroendocrine brain structures, the magnocellular cells of the SON and PVN, producing vasopressin and oxytocin, were analyzed for neuropathological changes (Table 1-2, Additional file 1). These neuroendocrine neurons contained neither poly(GA) nor pTDP-43 pathology (Fig. 3a-f, Table 2, Additional file 1). However, poly(GA) inclusions and pTDP-43 pathology were observed in the smaller neurons in between the magnocellular cells of the PVN (Fig. 3d,f).

## Discussion

Neuropathological assessments of brain regions associated with the circadian sleep/wake cycle are lacking in patients with ALS and/or FTLD. We now conducted a neuropathological study of the pineal gland and VIP-ir SCN-related neurons in *C9orf72-* and non*C9orf72-*related ALS and/or FTLD-TDP patients. In *C9orf72* cases, we observed numerous DPR pathological lesions in the melatonin-producing pinealocytes. On the other hand, pTDP-43 pathology was absent in the pineal gland of both *C9orf72* and non*C9orf72* cases. Although not statistically significant towards non*C9orf72* cases, the VIP-ir SCN-related neurons showed few poly(GA) inclusions in 50% of the *C9orf72* cases. No pTDP-43 pathology was observed in the SCN-related neurons of both *C9orf72* and non*C9orf72* cases. Besides, VIP-negative neurons present in the VIP-ir area showed DPR and/or pTDP-43 pathology. The abundant DPR pathology seemed to be specific to the neuroendocrine pineal gland, since other neuroendocrine brain structures (the magnocellular cells of SON and PVN) in the hypothalamus were unaffected. These magnocellular neuroendocrine hypothalamic nuclei were also spared from pTDP-43 pathology, confirming previously published data [11].

In previous studies, AD- and PD-related pathological lesions were mainly observed in the SCN rather than in the pineal gland [17, 32, 36]. This neuroanatomically differs compared to *C9orf72* cases, in which pinealocytes are housing a significant number of pathological DPR inclusions, whereas the SCN-related neurons are only affected in 50% of the *C9orf72* cases. Consequently, in *C9orf72* cases, mainly the executor, and to a lesser extent neurons related to the “central biological clock”, are affected by DPR pathology. Whether this explains why ALS patients show more subtle sleep abnormalities and do not display the same prominent circadian sleep/wake disturbances as AD and PD patients, remains to be investigated.

It has previously been shown that the DPR regional burden in post-mortem brain tissue did not correlate with neurodegeneration, while the neuroanatomical distribution of pTDP-43 pathology did [14, 26]. Nevertheless, DPRs were specifically present in pinealocytes as a neuroendocrine cell type and previously published findings show cellular dysfunction as a major outcome of DPR toxicity in many in vitro and in vivo models [4]. Therefore, we hypothesize that DPR pathology in the pinealocytes might lead to pinealocyte dysfunction, and mild DPR pathology in the SCN-related neurons could implicate disturbances along the melatonin-stimulating pathway. This hypothesis needs to be tested by comparing sleep disturbances among ALS and/or FTLD patients with and without the *C9orf72* repeat expansion. Moreover, it remains to be investigated whether DPR pathology may directly impair the melatonin-synthesizing and -secreting function of the pinealocytes. This could be done by e.g. determining serum and cerebrospinal fluid melatonin levels of ALS and/or FTLD patients with and without the *C9orf72* repeat expansion. Nonetheless, the morphological differences between the distinct neuroendocrine brain cells (pinealocytes versus magnocellular cells of SON and PVN) could also explain the specific appearance of DPR pathology in the pinealocytes, representing a harmless accumulation of these proteins rather than a functional alteration of the sleep/wake-associated cells. This explanation is in line with the abundant DPR pathology in cerebellar granule cells, without accompanying pTDP-43 inclusions and neurodegeneration [26].

Of note, DPR pathology does not exclusively affect neurons and has been shown before in the Sertoli cells [3], ependymal cells [35] and, more recently, in the skeletal muscle [10] of *C9orf72* patients. Pinealocytes are considered as a neuroendocrine cell type, without being real neurons. Therefore, the abundant DPR pathology in pinealocytes expands the non-neuronal spectrum of DPR pathology.

There are several limitations of our study. First, the cohort size in this study is small (especially for cases analyzed for the SCN-related neurons) as tissue availability was limited. The low number of cases could explain the lack of significance when comparing poly(GA) pathology in the SCN-related neurons of the *C9orf72* cases to the non*C9orf72* cases. However, the complete absence of DPRs in non*C9orf72* cases and the significant prevalence of DPRs in other brain regions of *C9orf72* cases, such as the pineal gland, argues in favor of *C9orf72*-related DPR expression in the SCN-related neurons. Second, pre-existing paraffin blocks of the hypothalamus were available covering only parts of this brain region. Therefore, the tissue was not suitable for stereological assessments. Consequently, we could not assess neuron loss in the investigated brain regions to observe a direct effect of DPR aggregates on neuronal viability. Third, clinical data on sleep disturbances were not collected for our patients and, therefore, we could not investigate the correlation between the neuropathological findings and clinical assessments of circadian sleep/wake disturbances. Finally, breathing abnormalities and muscle weakness will most likely still have the largest share in explaining sleep abnormalities of *C9orf72* ALS patients.

## Conclusions

We observed DPR, but no pTDP-43 pathology in the circadian sleep/wake-associated cells of ALS and/or FTLD-TDP patients. Abundant DPR pathological lesions in the pineal gland of *C9orf72* ALS and/or FTLD-TDP cases may indicate the involvement of pinealocyte dysfunction. Few poly(GA) inclusions observed in VIP-ir SCN-related neurons could implicate disturbances of the SCN-pineal gland axis in *C9orf72* cases. These neuropathological findings provide new insights in an underlying pathological correlative for the circadian sleep/wake disturbances, which might be involved in the disease course of ALS and/or FTLD-TDP patients carrying the *C9orf72* hexanucleotide repeat expansion. Further investigation on the circadian melatonin-producing and -secreting capacity of the pinealocytes, and the presence of circadian sleep/wake disturbances in *C9orf72* ALS and/or FTLD-TDP patients, is needed to clarify the functional impact of the DPR pathology in circadian sleep/wake-associated cells.

## Supplementary information

**Additional file 1:** List of human autopsy cases and neuropathological assessments per case

**Additional file 2: Figure S1.** Poly(GA) pathology in the pineal gland of *C9orf72* and non*C9orf72* ALS and FTLD cases. **Figure S2.** VIP-immunostaining of the SCN-related neurons in *C9orf72* and non*C9orf72* ALS and FTLD cases.

## Data Availability

Most data generated or analyzed during this study are included in this published article and in its supplementary information files. Additional data analyzed during the current study are available from the corresponding author upon reasonable request.

## Abbreviations

AD: Alzheimer’s disease
ALS: Amyotrophic lateral sclerosis
*C9orf72*: Chromosome 9 open reading frame 72
DAB: 3,3′-diaminobenzidine
DPR: Dipeptide repeat protein
FLTD-TDP: Frontotemporal lobar degeneration with TDP-43 pathology
FTD: Frontotemporal dementia
IQR: Interquartile range
Ir: Immunoreactive
NIA-AA: National Institute on Aging and Alzheimer’s Association
PD: Parkinson’s disease
pTDP-43: Phosphorylated transactive response DNA-binding protein 43 kDa
PVN: Paraventricular nucleus
RBD: Rapid eye movement sleep behavior disorder
SCN: Suprachiasmatic nucleus
SD: Standard deviation
SON: Supraoptic nucleus
VIP: Vasoactive intestinal peptide
