# Supplementary material for: Circadian sleep/wake-associated cells show dipeptide repeat protein aggregates in C9orf72-related ALS and FTLD cases
Source: Acta Neuropathol Commun. 2019 Dec 2;7:189. doi: 10.1186/s40478-019-0845-9 (PMC6889626; doi:10.1186/s40478-019-0845-9)
Supplement: Supplementary file 2 — Additional file 2: Figure S1. Poly(GA) pathology in the pineal gland of C9orf72 and nonC9orf72 ALS and FTLD cases. Figure S2. VIP-immunostaining of the SCN-related neurons in C9orf72 and nonC9orf72 ALS and FTLD cases. [file 40478_2019_845_MOESM2_ESM.pdf]

**Additional File 2**

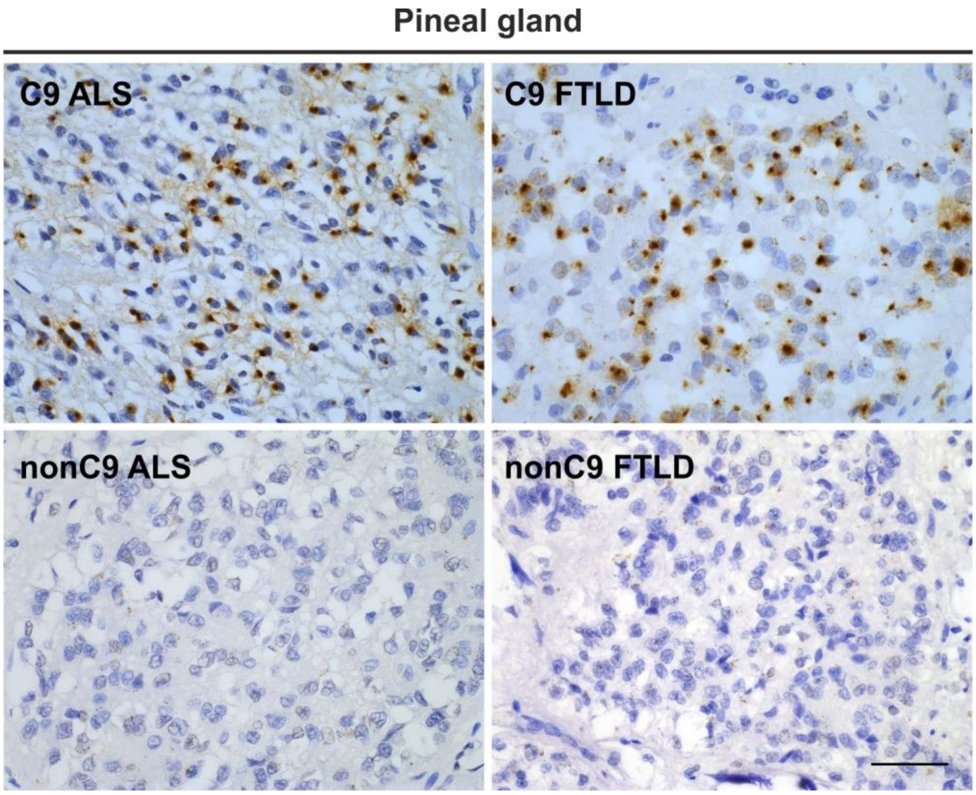


**Figure S1: Poly(GA) pathology in the pineal gland of *C9orf72* and non*C9orf72* ALS and FTLD cases.** Numerous poly(GA) inclusions were observed in the pineal gland of *C9orf72* cases with ALS or FTLD in a similar manner (case C9-2 and C9-7), but not in the pineal gland tissue of non*C9orf72* cases (case nonC9-11 and nonC9-1). Scale bar represents 50 µm.

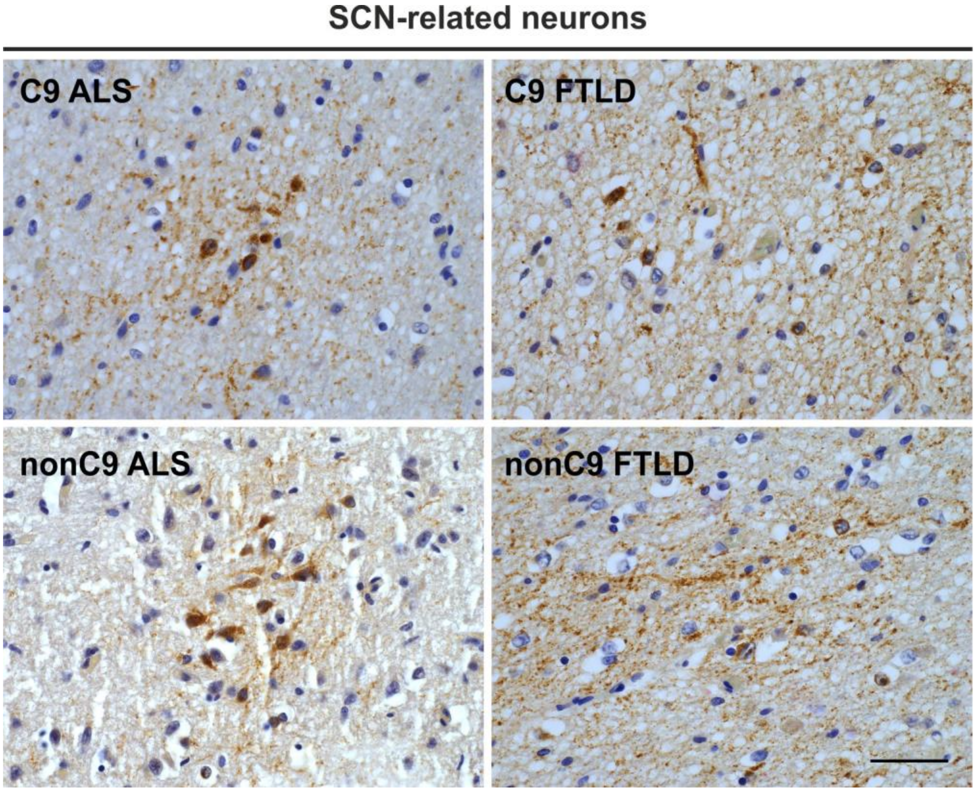


**Figure S2: VIP-immunostaining of the SCN-related neurons in *C9orf72* and non*C9orf72* ALS and FTLD cases.** VIP-positive neurons are observed in ALS or FTLD-TDP cases with and without the *C9orf72* repeat expansion (case C9-3, C9-7, nonC9-17, nonC9-1). Due to mild-moderate variations in the cutting levels of the hypothalamus sections, it is not possible to draw conclusions about differences in the pattern of the SCN-related neurons. Scale bar represents 50 µm. VIP stands for vasoactive intestinal peptide; SCN, suprachiasmatic nucleus
